# Simultaneous occurrence of medullary and papillary thyroid microcarcinomas: a case series and review of the literature

**DOI:** 10.1186/1752-1947-7-26

**Published:** 2013-01-21

**Authors:** Zaina Adnan, Eldad Arad, James Dana, Yaakov Shendler, Elzbieta Baron

**Affiliations:** 1 The Institute of Endocrinology, Zvulun Medical Center-Clalit Medical Services, Akko Str. 192, Kiryat Bialik, Israel; 2 The Catholic Medical Center, Manchester, NH 03102, USA; 3 The Institute of Pathology Carmel Medical Center, Haifa, Israel

**Keywords:** Calcitonin *RET* proto-oncogene, Medullary thyroid carcinoma, Papillary thyroid carcinoma, Papillary thyroid microcarcinoma

## Abstract

**Introduction:**

Papillary thyroid microcarcinoma has been demonstrated to present in association with medullary thyroid carcinoma, however, medullary thyroid carcinoma and papillary thyroid carcinoma represent rare entities. In recent years this rarity has been increasingly observed. The pathogenesis is still controversial. Genetic analysis of *RET* proto-oncogenes in cases of simultaneous papillary thyroid carcinoma and medullary thyroid carcinoma has so far provided conflicting results; although it seems that germline mutations play a potential role in the development of both histological types.

**Case presentations:**

This paper describes four rare cases of simultaneous medullary thyroid carcinoma and papillary thyroid microcarcinoma with unique features:

Case one was a 43-year-old Jewish woman, born in Israel, daughter of a Latvian immigrant mother and a father born in Israel. Case two was a 44-year-old Arab woman born in Israel. Case three was a 45-year-old Jewish woman, born in Israel, daughter of Moroccan immigrant parents and is unique for the presence of lymph node metastatic medullary thyroid carcinoma, and one lymph node with metastatic papillary carcinoma found in the same side. Case four was a 77-year-old Jewish woman, born in Iraq. These cases are unique in their composition of thyroid carcinoma, consisting of histologic features of medullary thyroid carcinoma, papillary thyroid microcarcinoma, and follicular thyroid adenoma. The four cases represent different ethnicity groups that live in north Israel, and case four is notable for the advanced age of the patient (77 years).

**Conclusion:**

These four cases add more data supporting the coincidental coexistence of papillary thyroid microcarcinoma and medullary thyroid carcinoma; our results may suggest that the simultaneous occurrence of medullary thyroid carcinoma and papillary thyroid microcarcinoma is generally a simple reflection of this coincidence. Endocrinologists and pathologists should be aware of this entity. The pathologist can play a pivotal role in identifying papillary thyroid microcarcinoma in concurrent existence with medullary thyroid carcinoma.

## Introduction

Papillary thyroid carcinoma (PTC), the most common type of thyroid carcinoma (75% to 80%), originates from follicular cells of the endoderm. PTC synthesizes thyroglobulin, organic iodine (I), and thyroid hormones. Medullary thyroid carcinoma (MTC) represents only 5% to 8% of thyroid carcinomas. It is derived from the parafollicular C-cell which originates in the ultimobranchial body of the neural crest. MTC synthesizes and secretes calcitonin and other neuroendocrine peptides [1].

The cell origin and histopathological features of PTC and MTC have been considered to differ. In the English literature, 71 cases of concurrent PTC and MTC have been reported. Cases with PTC and MTC presenting together in the same primary tumor, termed a ‘mixed medullary and follicular thyroid carcinoma’ (MMFTC), represent the simultaneous occurrence of rare and distinctly different entities. Lamberg *et al*. first reported MTC with PTC in 1981 [2]. In 2004, Biscolla *et al*. reported high rates of concurrent PTC (13.8%, 27 out of 196) in patients with MTC and, recently, in 2010, Kim *et al*. identified simultaneous PTC (19%, 10 out of 53) in patients with MTC [3,4]. These reported cases displayed well separated MTC and PTC, and there was a high tendency of papillary thyroid microcarcinoma (PTMC) with a tumor size less than or equal to one cm.

Our goal is to describe four additional cases of this rare entity, review the literature, and emphasize the important roles of the endocrinologist and pathologist in identifying new patients with this rare association.

## Cases presentations

### Case 1

A 43-year-old Jewish woman, born in Israel, the daughter of a Latvian immigrant mother and a father born in Israel, was referred to our medical center for multinodular goiter evaluation; there was no apparent family history of endocrine disorders or any previous external radiation therapy. Fine needle aspiration (FNA) disclosed a proliferative lesion suspicious for neoplasia. In 2005, the patient underwent a two-stage total thyroidectomy and right modified neck dissection for MTC. On macroscopic examination, the specimen revealed two separate tumors: the first one, a well-demarcated two cm maximal diameter lesion, and a second lesion measuring 1.2cm. The distance between the two masses was 0.9cm. Microscopy from the right lobe and isthmus disclosed medullary carcinoma measuring two cm in diameter. The tumor was composed of nests of oval cells with diffusely stippled nuclei staining positive for calcitonin and negative for thyroglobulin.

**Figure 1:**
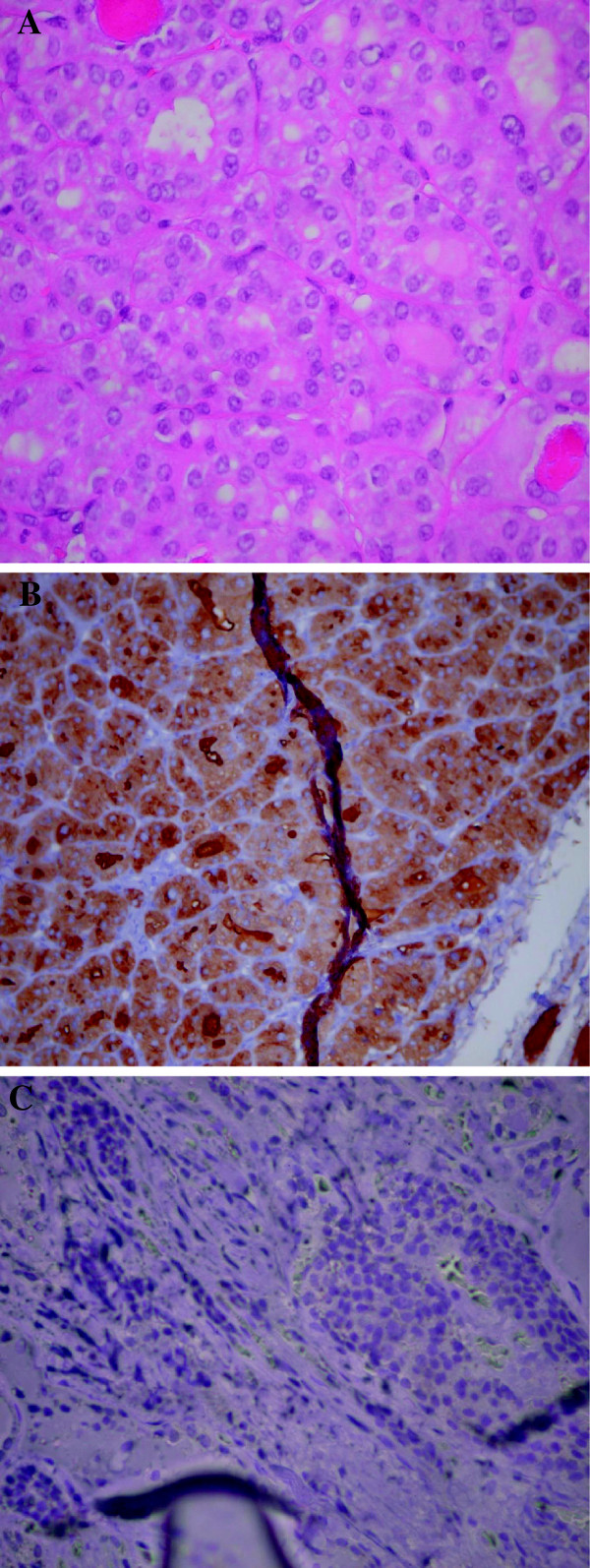
(A) Papillary thyroid carcinoma, hematoxylin and eosin staining, magnification × 400; (B) Positive thyroglobulin immunostaining for papillary thyroid carcinoma, magnification × 400; (C) Negative thyroglobulin immunostaining in medullary thyroid carcinoma, magnification × 200.

A 1.2cm in diameter follicular adenoma, with a microfollicular pattern, was partially circumscribed by a thin capsule. The tumor cells, with rounded nuclei, distinct nucleoli, and clumped chromatin, stained positive for thyroglobulin. Calcitonin staining showed a few small positive cell groups, but was otherwise negative. The non-neoplastic thyroid tissue revealed lymphocytic infiltration and occasional colloid nodules. The left thyroid lobe histology disclosed a tiny focus, two mm in size, consistent with micropapillary carcinoma (Figure 1). No lymph node metastasis was found. The patient’s presurgical calcitonin level (2170ng/L, normal value <13ng/L), carcinoembryonic antigen (CEA) level (51mg/L, normal reference range value less than five mg/L), and thyroid-stimulating hormone were within the normal range. The patient was treated surgically followed by I-131 100 mCi.

One year postsurgery, the patient’s calcitonin levels were less than five ng/L, and her thyroglobulin levels were no longer measurable. Point mutation for *RET* proto-oncogene was tested by deoxyribonucleic acid (DNA) analysis of the peripheral leukocytes and found to be negative (*RET* exon 8, 10, 11, 13–16 and *RET* gene mutation exon 5 were tested).

### Case 2

A 44-year-old Arab woman, born in Israel, was referred to our department for evaluation of a thyroid nodule. The presurgical FNA, taken from the right lobe, suggested follicular neoplasia. In October 2007, the patient underwent right thyroidectomy and isthmectomy. Analysis of the intraoperative frozen section disclosed the presence of carcinoma, with high suspicion of MTC, and subsequent total thyroidectomy was performed. The histology of the right lobe disclosed medullary carcinoma one cm in diameter, staining positively for calcitonin, chromogranin, synaptophysin, and partially for CEA. Congo red histochemical stain and polarized microscopy disclosed no evidence of amyloid. A tiny microscopic focus less than 0.1cm in diameter, consistent with PTMC, was documented. The left lobe histology revealed a 1.3cm nodule compatible with follicular adenoma. Following surgery, the patient underwent right modified neck dissection. No lymph node metastases were detected and I-131 100 mCi therapy was performed for remnant ablation. Point mutation of the *RET* proto-oncogene was found to be negative by DNA analysis of the peripheral leukocytes (*RET* exon 8, 10, 11, 13–16 and *RET* gene mutation exon 5 were tested).

### Case 3

A 45-year-old Jewish woman, born in Israel, the daughter of Moroccan immigrant parents was referred to our clinic for a multinodular goiter. Ultrasound examination of her neck showed a multinodular goiter with a very suspicious hypoechoic, not well demarcated, right lobe lesion measuring 1.2cm. The left lobe showed two nodules, 0.9cm and 0.6cm in size. The patient underwent ultrasonography (US)-guided FNA. The results were highly suspicious for follicular neoplasia. In August 2007, a total thyroidectomy was performed, which disclosed a 1.2cm diameter right medullary thyroid and multiple foci of papillary carcinoma with a maximal diameter of 0.4cm, both in the right lobe.

One month later, a chest computed tomography (CT) disclosed three small lung nodules up to 6mm in diameter.

Two months later, a right side modified neck dissection and central compartment dissection were performed. Histology revealed that 11 out of 30 lymph nodes had metastatic medullary carcinoma, levels IIA, IIB, III–IV, and one lymph node out of 10 had metastatic papillary carcinoma, level V–VI. External beam radiotherapy and I-131 remnant ablation were performed. Her calcitonin level, before her second operation, was 800ng/L, and 24-hour metanephrines and catecholamines were within normal range.

After her second operation, the patient underwent neck US, indium-111 somatostatin scan (Octreoscan™), positron emission tomography-CT and whole body scan with I-131, which ruled out further malignancy.

Her calcitonin level remains persistently slightly high (10.4ng/L), and her thyroglobulin level and thyroglobulin antibodies were undetectable under levothyroxine therapy.

Detection for point mutation of the *RET* proto-oncogen germline was negative by DNA polymerase chain reaction (PCR) analysis of peripheral leukocytes DNA (*RET* exon 8, 10, 11 13–16, and *RET* gene mutation exon 5 were tested).

### Case 4

A 77-year-old Jewish woman, born in Iraq, presented to our clinic for management of a right thyroid nodule discovered by neck CT that had been ordered due to cervical spine pain. She was under medical treatment for osteoporosis, for eight years, with alendronate. The patient denied any family history of endocrine disorder, thyroid carcinoma, or radiation exposure. Her thyroid US disclosed a solid thyroid nodule in the right lobe, 1.63cm in size, with peripheral hypervascularization. US-guided FNA diagnosed follicular neoplasia. The results of her preoperative thyroid function tests and calcium level were normal. In February 2011, the patient underwent surgery consisting of right side thyroidectomy and isthmectomy. The histology results showed a hyperplastic nodule with a small focus of 0.4cm of papillary carcinoma within a 1.7cm follicular adenoma. A 0.2cm focus of MTC was found a distance of 0.3cm from the inked thyroid surface. It stained positive for calcitonin, CEA and pankeratin. One month postoperatively, her calcitonin level was less than 0.2ng/L, CEA level 1.2 189mg/L (normal reference range value less than five mg/L), and her thyroglobulin, due to partial thyroidectomy, remained 39mcg/L. Her thyroid function tests, calcium level, and 24-hour metanephrines and catecholamines were all within the normal levels.

Detection for point mutation of the *RET* proto-oncogen germline was negative by DNA PCR analysis of peripheral leukocytes (*RET* exon 8, 10, 11 13, 14, 15, 16, and *RET* gene mutation exon 5 were tested).

The four cases are summarized in Table 1.

**Table 1:** Clinical characteristics of four patients with concurrent medullary thyroid carcinoma and papillary thyroid microcarcinoma

**No.**	**Age (years)**	**Sex**	**Pathology**	**Size**	**Laterality**	**Lymph no metastasis**
				**(cm)**		
1.	43	F	MTC	2.0	R	none
			FTA	1.2	R	
			PTMC	0.2	L	none
2.	44	F	MTC	1.0	R	none
			PTMC	0.1	R	none
			FTA	1.3	L	
3.	45	F	MTC	1.2	R	present
			PTMC	0.4	R	present
4.	77	F	MTC	0.3	R	none
			PTMC	0.4	R	none
			FTA	1.7	R	

*FTA* follicular thyroid adenoma; *L* left lobe; *MTC* medullary thyroid carcinoma; *PTMC* papillary thyroid microcarcinoma; *R* right lobe.

## Discussion

The simultaneous, discreet, existence of PTC and MTC, separated by normal tissue, is increasingly being identified. This entity differs from MMFTC, which is defined as a primary tumor showing morphological features of MTC together with immunoreactivity of calcitonin, and the morphological features of follicular carcinoma with reactivity to thyroglobulin [5].

Reviewing the English literature, about 77% (55 cases out of 71) of the papillary component presented as PTMC, including the cases presented in this manuscript (Table 2). PTMC is defined as PTC with a tumor size less than or equal to one cm at the greatest diameter. PTMC accounts for up to 30% of all PTC in some surgical series, and up to 35% of the general population displayed PTMC in autopsy series [6,7]. Similar to our results, Kim *et al.* identified PTMC in all their study patients.

**Table 2:** Literature review of the cases with concurrent medullary thyroid and papillary thyroid carcinoma

	**No. of cases**	**F:M**	**No. of micro:macro PTC**	Cervical LN Metastasis*
Case reports	30	19:11	20:10	16
Biscolla *et al*.	27	18:9	21:6	11
Kim *et al.*	10	9:1	10:0	4
Our cases	4	4:0	4:0	1

F female; M male; MTC medullary thyroid carcinoma; PTC papillary thyroid carcinoma; LN lymph node.

*MTC, PTC and PTC with MTC.

Based on our observations, we may suggest that most cases of concurrent MTC and PTMC are a coincidence. One possible explanation is that the sections were examined in too thin a slice to disclose these pathological abnormalities. The cases presented here were examined by the same pathologist at one location. Therefore, careful pathological examination represents a key point in disclosing this combination of pathology.

Other possible explanations for the simultaneous coexistence may be linked to the presence of *RET* proto-oncogene mutation in both papillary and medullary thyroid cells [8]. Sporadic MTC is associated with about 30% to 50% of cases with mutations in the *RET* proto-oncogene [9]. Point mutations and rearrangements of the tyrosine kinase receptors RET (ret/PTC) and NTRK1 have been characterized as specific for PTC, and have been documented in about 20% to 40% of cases [8-10]. A somatic point mutation of the *BRAF* gene has recently been identified as the most common genetic event in PTC [11-13].

The exact explanation for these rare simultaneous tumors is somewhat unclear. There are different theories regarding synchronous solid organ malignancies. One possible explanation is the ‘common stem cell theory’ as proposed by Ljungberg *et al*. [14]. These authors postulated that there might be a common progenitor cell, possibly in the ultimobranchial body, which undergoes divergent differentiation giving rise to both parafollicular and follicular cell lines.

This theory is supported by the presence of the same receptors and immunochemical markers in both tumors despite their different embryologic origins [14].

Another potential theory is a common tumorigenic stimulus such as radiation exposure that promotes the malignant transformation of both endodermal and neural crest-derived cell lines [15]. Genetic analysis of *RET* oncogene in cases of simultaneous PTC and MTC has so far provided conflicting results [16,17].

The more appealing hypothesis, reported in most studies, refers to a potential role of *RET* germline mutations in the development of both histological types.

## Conclusions

In the absence of a common background of the four cases presented here (the patients had different ethnicities and lived in different small cities in the northern part of Israel) and despite the limited number of patients, we may conclude that the majority of concurrent MTC and PTC, especially PTMC cases, might simply be coincidental, and related to a careful pathological examination.

Our findings are supported by previously published data. Endocrinologists and pathologists should keep in mind the possible coexistence of these entities.

## Consent

Written informed consents were obtained from the patients for publication of these case reports and any accompanying images. Copies of the written consents are available for review by the Editor-in-Chief of this journal.

## Abbreviations

CEA: Carcinoembryonic antigen; CT: Computed tomography; FNA: Fine needle aspiration; I: Iodine; MMFTC: Mixed medullary and follicular thyroid carcinoma; MTC: Medullary thyroid carcinoma; PCR: Polymerase chain reaction; PTC: Papillary thyroid carcinoma; PTMC: Papillary thyroid microcarcinoma; US: Ultrasonography.

## Competing interests

The authors declare that they have no competing interests.

## Authors’ contributions

ZA was the physician, drafted and edited the manuscript and obtained the patients’ informed consents. EA searched for previous relevant cases and reviewed the edited manuscript. JD participated in preparing the manuscript and checked spelling. YS performed the pathological material. BE was the attending physician in the out-patient clinic and the endocrinologist who attended the patients in hospital and took care of them during the entire period. All authors read and approved the final manuscript.
